# 

**Published:** 1846-10

**Authors:** 


### CONTENTS OF N° XLIV

OF THE

### British and Foreign Medical Review.

OCTOBER, 1846.

---

### Part First.—Analytical and Critical Reviews.

---

PAGE

Art. I.—The Nature and Treatment of Cancer. By W. H. Walshe, m.d., Professor of Pathological Anatomy in University College, Physician to University College Hospital, and to the Hospital for Consumption and Diseases of the Chest . . . . . . . 293

Cancer of the Digestive Organs and Appendages . . . ib.
Cancer of the Respiratory Organs and Appendages . . . 300
Cancer of the Kidney . . . . . . 303
Cancer of the Bladder . . . . . . 305
Cancer of the Genital Organs . . . . . . 306
Cancer of the Organs of Innervation and Appendages . . . 309

Art. II.—Die Gallige Dyscrasie (Icterus) mit acuter gelber Atrophe der Leber. Von Dr. P. J. Horaczek, practischen Arze in Wien, &c. . . 311

On Bilious Dyscrasy (Icterus) with Acute Yellow Atrophy of the Liver. By Dr. P. J. Horaczek.

Art. III.—Transactions of the Provincial Medical and Surgical Association. New Series. Vol. II . . . . . . 319

Mr. Crosse's History of the Cure of a Tumour . . . ib.
Dr. Favell on Grinders' Asthma . . . . . 320

Art. IV.—1. Pathologische Anatomie des menslichen Körpers. Von Julius Vogel. Erste Abtheilung. (Allgemeiner Thiel.) . . . . 323

The Pathological Anatomy of the Human Body. By Julius Vogel.

§

PAGE

2. JULII VOGEL Icones Histologiæ Pathologicæ. Tabulæ Histologiam Pathologicam illustrantes. Viginti sex Tabulæ, continentes CCXCI Figuras, quarum CCLXX ad naturam delineatæ sunt . . . . 323

Erläuterungstafeln zur pathologischen Histologie mit vorzüglicher Rücksicht auf sein Handbuch der pathologischen Anatomie, herausgegeben von Dr. JULIUS VOGEL, ausserordentl. Professor der Medizin in Göttingen. Sechs und zwanzig Tafeln mit 291 Figuren, wovon 270 nach der Natur gezeichnet sind.

Illustrations of Pathological Histology, illustrative of his Manual of Pathological Anatomy. By Dr. JULIUS VOGEL, Extraordinary Professor of Medicine at Göttingen.

ART. V.—Geschichte der Findlinge in Oesterreich mit besonderer Rücksicht auf ihre Verhältnisse in Illyrien. Von Dr. RAIMUND MELZER, K.k. Director der Staats-und Local-Wohlthätigkeits-Anstalten zu Laibach . . 338

History of Foundlings in Austria, with especial Reference to their Condition in Illyria. By Dr. RAIMUND MELZER, Imperial Director of the State and Local Charitable Institutions of Laybach.

ART. VI.—Observations and Essays on the Statistics of Insanity; including an inquiry into the Causes influencing the Results of Treatment in Establishments for the Insane: to which are added the Statistics of the Retreat, near York. By JOHN THURNAM, Licentiate of the Royal College of Physicians of London, Resident Medical Superintendent of the Retreat, near York . . 349

On the Statistics of Insanity in general . . . . ib.

Essays on the Liability to Insanity . . . . 360

Historical Sketch and Statistics of the Retreat . . . 363

ART. VII.—1. Practical Surgery. By Robert LISTON. Fourth edition . 366

2. Lectures on the Operations of Surgery, and on Diseases and Accidents requiring Operations. By ROBERT LISTON, Esq., F.R.S.; with numerous Additions, by THOMAS D. MÜTTER, M.D., Professor of Surgery in Jefferson College, Philadelphia, &c. . . . . . 379

ART. VIII.—Die Acute Entzündung der Serösen Häute der Gehirns und Rückenmarks. Nach eigenen Beobachtungen am Krankenbett geschrieben. Von Dr. JOSEPH NEISSER, Prakt. Arzt zu Berlin . . . 384

Acute Inflammation of the Serous Membranes of the Brain and Spinal Cord; described from Personal Observation at the Bedside. By Dr. JOSEPH NEISSER, Physician at Berlin.

ART. IX.—On Disorders of the Cerebral Circulation, and on the Connexion between Affections of the Brain and Diseases of the Heart. By GEORGE BURROWS, M.D., late Fellow of Caius College, Cambridge, Fellow of the Royal College of Physicians, London, Physician and Lecturer on the Principles and Practice of Medicine at St. Bartholomew's Hospital . . . 408

ART. X.—1. Examen Clinique de l'Hydrothérapie. Par H. E. SCHEDEL, Docteur en Médecine . . . . . . . . 428

PAGE

2. The Dangers of the Water Cure, and its Efficacy examined. By JAMES WILSON, M.D., and JAMES M. GULLY, M.D. . . . 428

3. The Cold Water Cure: its Use and Misuse examined. By HERBERT MAYO, M.D., F.R.S., formerly Surgeon of Middlesex Hospital . . . ib.

4. A Medical Visit to Graeffenberg. By Sir CHARLES SCUDAMORE, M.D., F.R.S. . . . . . . . ib.

5. Hydropathy. By EDWARD JOHNSON, M.D. . . . ib.

6. Graeffenberg; or, a True Report of the Water Cure, with an Account of its Antiquity. By ROBERT HAY GRAHAM, M.D. . . . ib.

7. Life at the Water Cure, or a Month at Malvern. By R. J. LANE . ib.

8. Confessions of a Water Patient. By Sir E. B. LYTTON, Bart. . ib.

ART. XI.—Redogörelse Sjukvärden ä Kongl. Seraphimer Lazarettets Afdelning för Invertes Sjuke, under Loppet af Ar 1842 . . . . 459

Medical Report of the Internal Diseases treated in the Royal Seraphim Hospital in Stockholm during the year 1842. By Dr. MAGNUS HUSS.

ART. XII.—Mesmerism in India, and its practical Application in Surgery and Medicine. By JAMES ESDAILE, M.D. . . . . 475

ART. XIII.—The Brain and its Physiology; a Critical Disquisition on the Methods of determining the Relations subsisting between the Structure and Functions of the Encephalon. By DANIEL NOBLE, M.R.C.S. . . . 488

ART. XIV.—On the Injurious Effects of Mineral Poisons in the Practice of Medicine, &c. &c. By HORATIO PRATER, M.D., PH.D. &c. . . . 545

ART. XV.—A Practical Treatise on the Diseases of Children. By JAMES MILMAN COLEY, M.D., Member of the Royal College of Physicians in London, &c. . 548

### PART SECOND.—Bibliographical Notices.

ART. I.—Notes and Recollections of a Professional Life. By the late WILLIAM FERGUSSON, M.D., Inspector-General of Military Hospitals. Edited by his Son JAMES FERGUSSON . . . . . . . 551.

ART. II.—The Microscopic Anatomy of the Human Body in Health and Disease, illustrated with numerous Drawings in Colour. By ARTHUR HILL HASSALL, F.L.S., M.R.C.S., &c. Parts I and II . . . . 552

ART. III.—Elements of Physics. By C. F. PESCHEL, Principal of the Royal Military College at Dresden, &c. Translated from the German, with Notes, by E. WEST . . . . . . . 553

PAGE

ART. IV.—The Vegetable Kingdom; or the Structure, Classification, and Uses of Plants, illustrated upon the Natural System. By JOHN LINDLEY, F.R.S., F.L.S., Professor of Botany in the University of London, and in the Royal Institution of Great Britain . . . . . . 554

ART. V.—The Anatomy of the Arteries of the Human Body, and its Applications to Pathology and Operative Surgery; with a series of Lithographic Drawings the Size of Nature. By RICHARD QUAIN, F.R.S. The Drawings by JOSEPH MACLISE, Esq., Surgeon . . . . . . 556

---

### PART THIRD.—Natural History and Treatment of Diseases.

---

I.—On Excessive Trust in Nature in the Cure of Diseases. By J. A. SYMONDS, M.D., Consulting Physician to the General Hospital, Bristol . . . 557

II.—On the Present State of Homœopathy in Germany. By Dr. A. MÜHRY, of Hanover . . . . . . . 565

III.—Report on the Homœopathic Treatment of Acute Diseases in Dr. Fleischmann's Hospital, during the months of May, June, and July, 1846. By GEORGE W. BALFOUR, M.D. Edin. . . . . . 567

Typhus Fever . . . . . . . 573

Febris Gastrica . . . . . . 576

Intermittent Fever . . . . . . ib.

Rheumatism . . . . . . 577

Diarrhea . . . . . . . 579

Dysentery . . . . . . . ib.

Cholera . . . . . . . 580

Colica . . . . . . . ib.

Scarlatina . . . . . . . 581

Pneumonia . . . . . . . ib.

Other Diseases . . . . . . 591

Remarks by the Editor . . . . . . 592

---

Books received for Review . . . . . . 594

INDEX, TITLE, &c.

